# Rising heavy precipitation extremes in Central European river basins under a high emission scenario

**DOI:** 10.1038/s41598-026-45624-9

**Published:** 2026-04-01

**Authors:** Mohammad Reza Eini, Haniyeh Salmani, Pouya Ghezelayagh, Mehdi Nodeh, Mikołaj Piniewski

**Affiliations:** 1 Department of Computational Landscape Ecology, Helmholtz Centre for Environmental Research GmbH - UFZ, Leipzig, Germany; 2 Department of Hydrology, Meteorology, and Water Management, Institute of Environmental Engineering, Warsaw University of Life Sciences – SGGW, Warsaw, Poland; 3 University of Alcalá, Madrid, Spain; 4 Economic and Institutional Analysis Group, IMDEA Water, Madrid, Spain; 5 Centre for Climate Research, Warsaw University of Life Sciences – SGGW, Warsaw, Poland; 6 Leibniz Universität Hannover, Hannover, Germany

**Keywords:** Flood, Poland, Central EU, Global warming, Trend, Climate sciences, Hydrology, Natural hazards

## Abstract

This study assesses historical trends and future projections of extreme precipitation events in Central Europe’s Vistula and Oder (Odra) transboundary river basins using climate indices recommended by the Expert Team on Climate Change Detection and Indices (ETCCDI). Utilizing bias-corrected EURO-CORDEX regional climate models under the high-emission RCP8.5 scenario, we evaluated changes in the frequency, intensity, and contribution of heavy precipitation events from 2006 to 2100 compared to a historical baseline (1990–2019). The findings demonstrate a consistent and statistically significant (based on Mann–Kendall and Sen’s slope) increase in extreme precipitation events, notably in heavy rainfall days (R10mm and R20mm), intensity metrics (maximum 1-day and 5-day precipitation, Rx1day and Rx5day), and the proportion of total precipitation contributed by extreme events (R95pTOT and R99pTOT). Spatial analysis reveals that the southern mountainous areas historically experience and will continue to experience the most pronounced changes, though significant increases are projected broadly across the region. Duration indices, such as consecutive dry and wet days (CDD, CWD), showed less clear and more uncertain future trends. The projected intensification of extreme rainfall events has important implications for flood risk management, infrastructure design, and water resource planning in Central Europe. These results underscore the urgent need for proactive adaptation measures to reduce vulnerabilities and enhance resilience against climate-induced precipitation extremes.

## Introduction

Recent observations indicate that the Earth’s climate is undergoing significant changes. Global temperatures are increasing, precipitation patterns are shifting, and extreme weather events, such as floods and droughts, are becoming more frequent and severe, while glaciers continue to recede^1–5^. These trends confirm that climate change is already in progress rather than being solely a future projection^6–10^. Extreme weather events, particularly changes in their frequency and intensity, often have a greater impact on ecological cycles, infrastructure, and human safety than shifts in climate averages. Therefore, comprehensive data on extreme events is essential for decision-makers and policy-makers responsible for planning in various climate-sensitive sectors, particularly as events formerly classified as extreme become increasingly ordinary in a changing climate^11^.

According to the World Health Organization (WHO), climate change represents one of the most pressing challenges of our time due to its impacts on human populations, economic systems, and natural ecosystems^7,10^. Recent studies in the Baltic Sea region and Central Europe further highlight the growing urgency of these challenges^12^.

A primary concern is the rising frequency and intensity of extreme weather events. There is mounting evidence of increased heatwaves, more hot days, prolonged dry spells, and intense rainfall events capable of causing severe flooding^3,13–16^. Projections suggest that extreme rainfall events will become even more common worldwide, potentially triggering major natural disasters. Such information is critical for the design and maintenance of hydraulic infrastructure, including flood barriers, dams, and water distribution systems, and for optimizing agricultural practices and enhancing environmental protection^10,11^.

To facilitate the monitoring of these changes, the Expert Team on Climate Change Detection and Indices (ETCCDI) has developed a set of indicators that quantify various aspects of extreme weather^17^. These indicators assess the frequency, intensity, spatial extent, duration, and timing of extreme events. The Intergovernmental Panel on Climate Change (IPCC) has cautioned that these alterations in climate parameters may lead to unprecedented weather phenomena^7^. Investigations on extreme weather events and indicators, and their trends, are critical subjects among scientists in determining and verifying climate change in an area of interest^11^.

Recent research shows that not only are these extreme events occurring more frequently than in the past, but their trends and spatial patterns have also changed significantly. For example, climate change caused changes in monsoon events in India, and more significant changes were detected over arid-cold regions of India than in other regions^4,10^.

Based on several research papers, Central Europe has experienced numerous extreme events in recent decades, and, at the same time, more floods and droughts happen during the year, especially over the warm season (June to September), and these changes could become more frequent in the future^12–14,16,18–21^. For instance, in September 2024 in Poland, precipitation exceeded 400 mm over three days, a new record for extreme events (https://hydrobim.pl/en/flood-in-poland-2024/)^22^. On the other hand, from 1 May to 30 June 2024, Poland’s average Climatic Water Balance (CWB) value was -121 mm, meaning that the total surface evaporation over Poland was 121 mm higher than the precipitation (https://susza.iung.pulawy.pl/en/komentarz/2024,05/). Extremely low CWB values are a symptom of agricultural droughts that frequently evolve into extremely low flows or hydrological droughts, which are increasingly common in Central Europe^13,23^. All studies indicate that this region requires further investigation and assessment, particularly regarding projections of extreme events.

Therefore, this study aims to analyze historical trends and future projections of extreme precipitation utilizing ETCCDI indices and EURO-CORDEX RCP8.5 datasets applied to two principal transboundary river basins in Central Europe and Poland (Oder/Odra and Vistula River Basins) within the Baltic Sea region. The research is driven by the availability of recently bias-corrected datasets and the necessity for a consistent assessment of extreme precipitation indicators.

## Materials and methods

In this section, the study area, the datasets used, and the extreme precipitation indicators are introduced.

### Study area

**Fig. 1:**
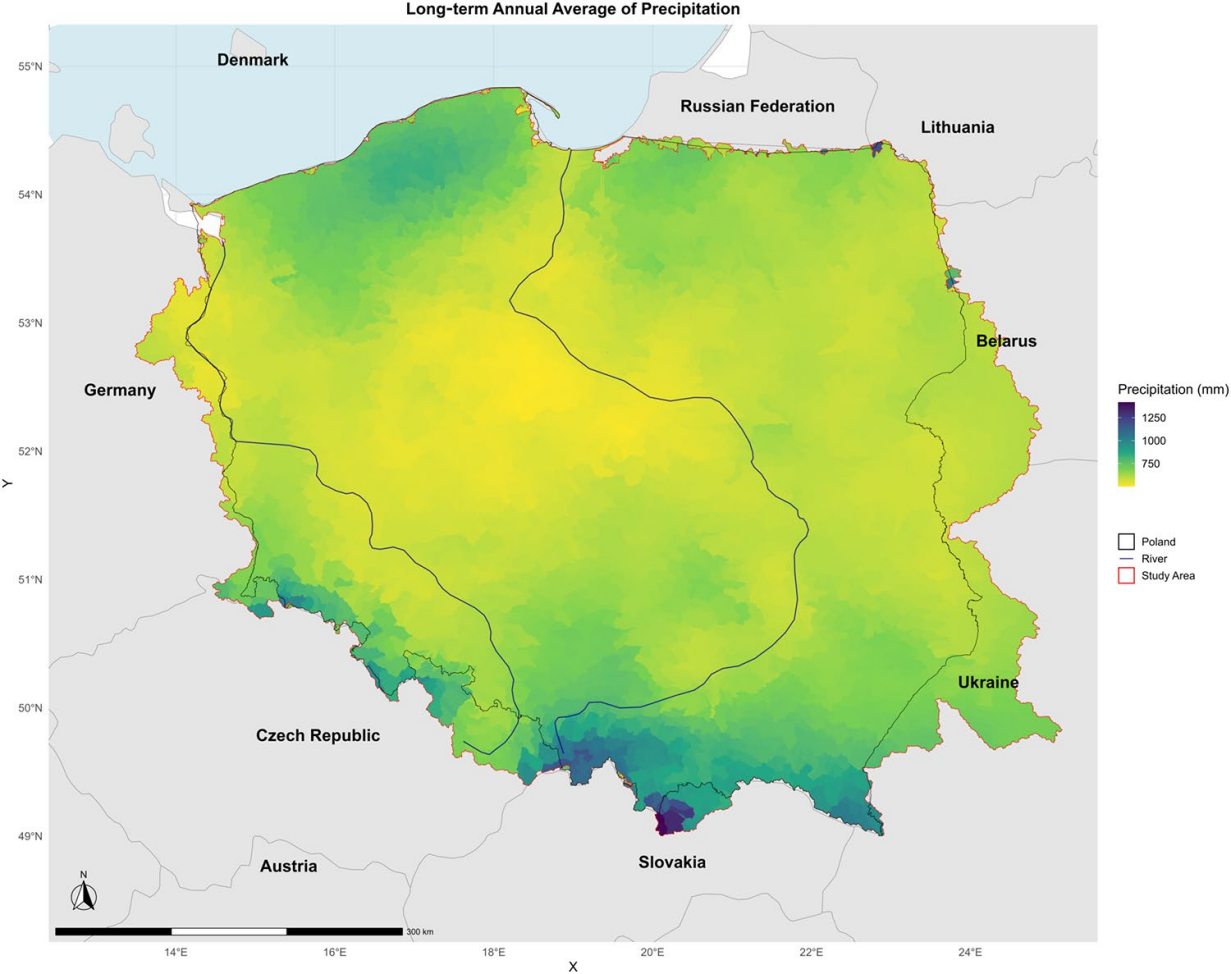
Study area and long-term annual average of precipitation over the study area.

In this research, we focused on the union of two large transboundary river basins (Oder/Odra and Vistula River Basins) and Poland in the Baltic Sea region and their union with Polish territory (Fig. 1). The research area, covering 349,766 km^2^, encompasses the territory of Poland (312,683 km^2^), as well as the source regions of the Vistula and Odra basins (37,083 km^2^). This transboundary study area was previously used in a series of publications on extreme precipitation^17^, drought ^18,21^ and water balance simulations^24^. According to Fig. 1, the southern part of this area receives the highest annual precipitation (approximately 1000 mm), while the central and lowland parts receive the lowest (400–500 mm). The average annual precipitation over the region is approximately 600 mm.

This region is the home of around 40 million people. Its geography is diverse, featuring extensive plains in the north and central regions, upland areas in the central part, and mountain ranges in the south, most notably the Carpathians and the Sudetes. This variety in terrain supports a range of natural landscapes, from lowland agricultural fields and forests to rugged highlands and mountainous regions. The region’s climate is generally temperate, characterized by a mix of oceanic and continental influences. Winters tend to be cold with regular snowfall, particularly in the mountainous and northeastern areas. At the same time, summers are typically warm, with more significant heat experienced in the southern and eastern parts. Precipitation is relatively evenly distributed throughout the year, although it can vary by region. The northern coastal areas experience more maritime conditions, whereas the interior regions are subject to more pronounced seasonal temperature differences. Overall, the region’s varied geography and climate play an important role in its natural environment, agricultural practices, and weather extremes and climate change challenges.

### Climate models and regional datasets

The daily precipitation data, with a resolution of 12.5 km, were obtained from six bias-corrected GCM-RCM (General Circulation Model-Regional Climate Model), as summarized in Table 1. These models were selected based on their availability in the EURO-CORDEX archive and their proven performance in reproducing climatological patterns over Central Europe in previous validation studies^24^. Utilizing an ensemble of six models allows for the assessment of inter-model variability, which serves as a proxy for projection uncertainty. While individual models may differ in their physics and parameterization schemes, the ensemble approach provides a more robust signal of future changes than any single model.

**Table 1:** Used datasets for precipitation projections.

Model	Global model	Regional model	RCM version	Model run scenario
A	CNRM-CM5	CNRM-ALADIN63	v2	r1i1p1
B	ICHEC-EC-EARTH	DMI-HIRHAM5	v2	r3i1p1
C	ICHEC-EC-EARTH	KNMI-RACMO22E	v1	r12i1p1
D	ICHEC-EC-EARTH	KNMI-RACMO22E	v1	r1i1p1
E	ICHEC-EC-EARTH	SMHI-RCA4	v1	r12i1p1
F	MPI-M-MPI-ESM-LR	SMHI- RCA4	v1a	r1i1p1

The simulations covered the period from 2006 to 2100 under the RCP8.5 scenario. Although the historical baseline is defined as 1990–2019 to capture recent climate conditions, the model projections begin in 2006. For the overlap period (2006–2019), the model data from the RCP8.5 runs were used to ensure a continuous and consistent future dataset, while the historical observational data (G2DC-PL+) were used solely for the retrospective trend analysis.

The study focused exclusively on the high-end emission scenario, RCP8.5. All GCMs were corrected using the quantile-mapping method. The historical baseline was defined as the 30-year period from 1990 to 2019 to ensure statistical robustness, particularly for the analysis of rare extreme events, adhering to WMO guidelines for climatological normals. The future projections utilize the EURO-CORDEX RCP8.5 simulations, which commence in 2006 following the CMIP5 protocol. Although this results in a temporal overlap (2006–2019) between the historical baseline and the start of the projection scenarios, utilizing the full 2006–2100 period is essential to capture near-term climate evolution. Restricting the historical baseline to the pre-2006 period (e.g., 1990–2005) would yield an insufficient sample size for detecting robust trends in hydrological extremes.

The reader is referred to the study by Marcinkowski et al.^24^ for more details on the climate scenarios and bias-correction procedure. This climate scenario data set under RCP8.5 has also been used in a recent study on future drought projections over Poland^21^.

The historical dataset (G2DC-PL +) is a gridded 2 km daily climate dataset for the union of the Polish territory and the Vistula and Odra basins and covers the 1951–2019 period. It is based on observed stations and developed by a geostatistical interpolation method (kriging)^25^. This dataset is used for hydrological modeling and climatic investigations in several studies^14,16–18^.

### ETCCDI indicators

Numerous indices are recommended to illustrate extreme precipitation events, varying from the intensity of single-event indicators to aggregated event indicators^26,27^. This study utilizes ten intensity-based precipitation indices suggested by the ETCCDI, described in Table 2. More info about these indicators can be found at http://etccdi.pacificclimate.org. These extreme rainfall indices have been used to identify the non-stationary properties of extreme rainfall events^28^. The Climate Data Tools (CDT) package in R was used for calculations (https://github.com/rijaf-iri/CDT).

**Table 2:** Description of ETCCDI precipitation indices used in the study.

Indicator	Description	Units
CDD	Maximum Consecutive Dry Days: Maximum number of consecutive days with RR < 1 mm	days
CWD	Maximum Consecutive Wet Days: Maximum number of consecutive days with RR > = 1 mm	days
PRCPTOT	Total Wet-day Precipitation: Total annual precipitation on wet days (RR > = 1 mm)	mm
R10mm	Number of Heavy Precipitation Days: Annual count of days with RR > = 10 mm	days
R20mm	Number of Very Heavy Precipitation Days: Annual count of days with RR > = 20 mm	days
R95pTOT	Precipitation Fraction from Very Wet Days: Contribution to total precipitation from days > 95th percentile	%
R99pTOT	Precipitation Fraction from Extremely Wet Days: Contribution to total precipitation from days > 99th percentile	%
Rx1day	Maximum 1-day Precipitation: Highest precipitation amount in a single day	mm
Rx5day	Maximum 5-day Precipitation: Highest precipitation amount in a 5-day period	mm
SDII	Simple Daily Intensity Index: Average precipitation amount on wet days (PRCPTOT / number of wet days)	mm/day

Note: RR refers to daily precipitation amount. Percentiles are calculated based on wet days during a reference period.

### Statistical methods

Non-parametric methods were employed to assess the presence and magnitude of trends in the precipitation indices over time. The Mann–Kendall (MK) test^29,30^ was used to detect the presence of monotonic trends (increasing or decreasing), and Sen’s slope estimation^30,31^ was used to quantify the magnitude of the linear trend (rate of change per year). These tests were applied separately to the annual historical time series (1990–2019) (aggregated from daily precipitation datasets) and the median projected annual time series from the model ensemble (2006–2100) under the RCP8.5 scenario.

The statistical analysis was conducted at two spatial scales:

1. *Grid-based analysis:* The MK test and Sen’s slope were calculated for each individual pixel (12.5 km resolution) to generate spatial maps of trend significance and magnitude across the study area.
2. *Regional analysis:* Spatially averaged time series were aggregated for the entire domain to assess general trends.

To address the issue of serial correlation (autocorrelation) in the time series, which can increase the probability of detecting false trends, a pre-whitening procedure was applied to the data prior to the MK test (Yue et al. 2002). These methods have been widely applied in recent hydrological studies for trend detection in multi-source rainfall data^32^. A significance level of p < 0.05 was used to determine statistical significance.

## Results

This section presents the analysis of historical trends and future projections for eleven precipitation-related indices defined by the Expert Team on Climate Change Detection and Indices (ETCCDI). The analysis uses historical gridded climate data (G2DC-PL +) covering 1990–2019 and an ensemble of six bias-corrected EURO-CORDEX regional climate model simulations driven by different global climate models under the high-emission RCP8.5 scenario for the period 2006–2100.

### Trends in extreme precipitation indicators

According to Table 3, for the future projections under RCP8.5, there are highly statistically significant increasing trends for nearly all indices related to precipitation amount (PRCPTOT), intensity (SDII, Rx1day, Rx5day), frequency of heavy events (R10mm, R20mm, R25mm), and the contribution of extremes to the total (R95pTOT, R99pTOT). The only indices without significant projected trends are those related to the duration of dry (CDD) and wet (CWD) spells, although CWD showed a tendency towards increasing.

**Table 3:** Non-parametric trend analysis for domain-averaged ETCCDI indicators.

Indicator	Source	Method	Statistic	p_value	Interpretation
CDD	Projections	Mann–Kendall	0.041 ↑	0.566	No considerable trend in the length of the most extended dry spells was detected in the future projections
CDD	Projections	Sen’s Slope	0.006 ↑		Negligible increase of about 0.006 days per year
CDD	Historical	Mann–Kendall	-0.053 ↓	0.695	No considerable trend in the length of the most extended dry spells was detected in the historical period
CDD	Historical	Sen’s Slope	-0.025 ↓		Slight decrease of about 0.025 days per year
CWD	Projections	Mann–Kendall	0.127 ↑	0.073	Weak evidence for a potential slight increase in the length of the most extended wet spells in future projections
CWD	Projections	Sen’s Slope	0.003 ↑		Slight increase of about 0.003 days per year
CWD	Historical	Mann–Kendall	-0.034 ↓	0.803	No significant trend has been found historically or definitively projected
CWD	Historical	Sen’s Slope	-0.005 ↓		Negligible decrease of about 0.005 days per year
PRCPTOT	Projections	Mann–Kendall	0.333 ↑	0.000	Total annual precipitation shows a significant increase in future projections
PRCPTOT	Projections	Sen’s Slope	0.964 ↑		Increase of approximately 0.96 mm per year
PRCPTOT	Historical	Mann–Kendall	0.177 ↑	0.175	Historical data also showed an increasing tendency, it did not reach statistical significance over the observed period
PRCPTOT	Historical	Sen’s Slope	2.223 ↑		Increase of about 2.22 mm per year over the historical period
R10mm	Projections	Mann–Kendall	0.396 ↑	**0.000**	Frequency of days with > = 10 mm of rain projected to continue and strengthen significantly in the future
R10mm	Projections	Sen’s Slope	0.043 ↑		Increase of about 0.043 days per year
R10mm	Historical	Mann–Kendall	0.310 ↑	**0.017**	Frequency of days with > = 10 mm of rain showed a significant increase historically
R10mm	Historical	Sen’s Slope	0.135 ↑		Increase of about 0.135 days per year over the historical period
R20mm	Projections	Mann–Kendall	0.470 ↑	**0.000**	Days with > = 20 mm of rain show a significant increasing trend in future projections
R20mm	Projections	Sen’s Slope	0.016 ↑		Increase of about 0.016 days per year
R20mm	Historical	Mann–Kendall	0.218 ↑	0.094	Historical data showed a rising tendency toward significance
R20mm	Historical	Sen’s Slope	0.035 ↑		Increase of about 0.035 days per year historically
R95pTOT	Projections	Mann–Kendall	0.481 ↑	**0.000**	Fraction of total rainfall from very wet days (above 95th percentile) is projected to increase significantly
R95pTOT	Projections	Sen’s Slope	0.664 ↑		Increase of about 0.66 percentage points per year
R95pTOT	Historical	Mann–Kendall	0.241 ↑	0.064	A similar, marginally significant increasing trend was observed historically
R95pTOT	Historical	Sen’s Slope	1.766 ↑		Increase of about 1.77 percentage points per year historically
R99pTOT	Projections	Mann–Kendall	0.535 ↑	**0.000**	A significant increasing trend in future projections
R99pTOT	Projections	Sen’s Slope	0.334 ↑		Increase of about 0.33 percentage points per year
R99pTOT	Historical	Mann–Kendall	0.140 ↑	0.284	No significant trend was detected in the historical data for this index
R99pTOT	Historical	Sen’s Slope	0.416 ↑		Increase of about 0.42 percentage points per year historically
Rx1day	Projections	Mann–Kendall	0.568 ↑	**0.000**	Intensity of the heaviest single-day rainfall event each year is projected to increase significantly
Rx1day	Projections	Sen’s Slope	0.086 ↑		Annual maximum 1-day rainfall increase of about 0.086 mm per year
Rx1day	Historical	Mann–Kendall	0.062 ↑	0.643	No significant trend was detected in the historical data
Rx1day	Historical	Sen’s Slope	0.068 ↑		Slight increase of about 0.068 mm annually
Rx5day	Projections	Mann–Kendall	0.418 ↑	**0.000**	Intensity of the heaviest 5-day rainfall event each year is projected to increase significantly
Rx5day	Projections	Sen’s Slope	0.117 ↑		Increase in the annual maximum 5-day rainfall total of about 0.117 mm per year
Rx5day	Historical	Mann–Kendall	0.090 ↑	0.498	No significant trend was detected in the historical data
Rx5day	Historical	Sen’s Slope	0.162 ↑		Increase of about 0.162 mm per year historically
SDII	Projections	Mann–Kendall	0.544 ↑	**0.000**	Average rainfall intensity on wet days projected to continue and strengthen significantly
SDII	Projections	Sen’s Slope	0.007 ↑		Increase in average wet-day intensity of about 0.0075 mm/day per year
SDII	Historical	Mann–Kendall	0.352 ↑	**0.007**	Average rainfall intensity on wet days significantly increased historically
SDII	Historical	Sen’s Slope	0.020 ↑		Increase of about 0.020 mm/day per year historically

bold numbers indicate statistically significant

Historically, significant increasing trends were detected for the frequency of heavy rainfall days (R10mm) and the average intensity on wet days (SDII). Several other indices (R20mm, R25mm, R95pTOT) showed increasing tendencies that approached or were marginally significant. However, key intensity metrics like Rx1day and Rx5day, total precipitation (PRCPTOT), and the contribution from the most extreme events (R99pTOT) did not show statistically significant trends over the historical period 1990–2019 for this region, despite the significant increases projected for the future. No significant historical trends were detected for the duration indices (CDD and CWD), suggesting that changes in the persistence of dry or wet spells have not yet emerged in the observational record.

### Temporal distribution of ETCCDI indicators

**Fig. 2:**
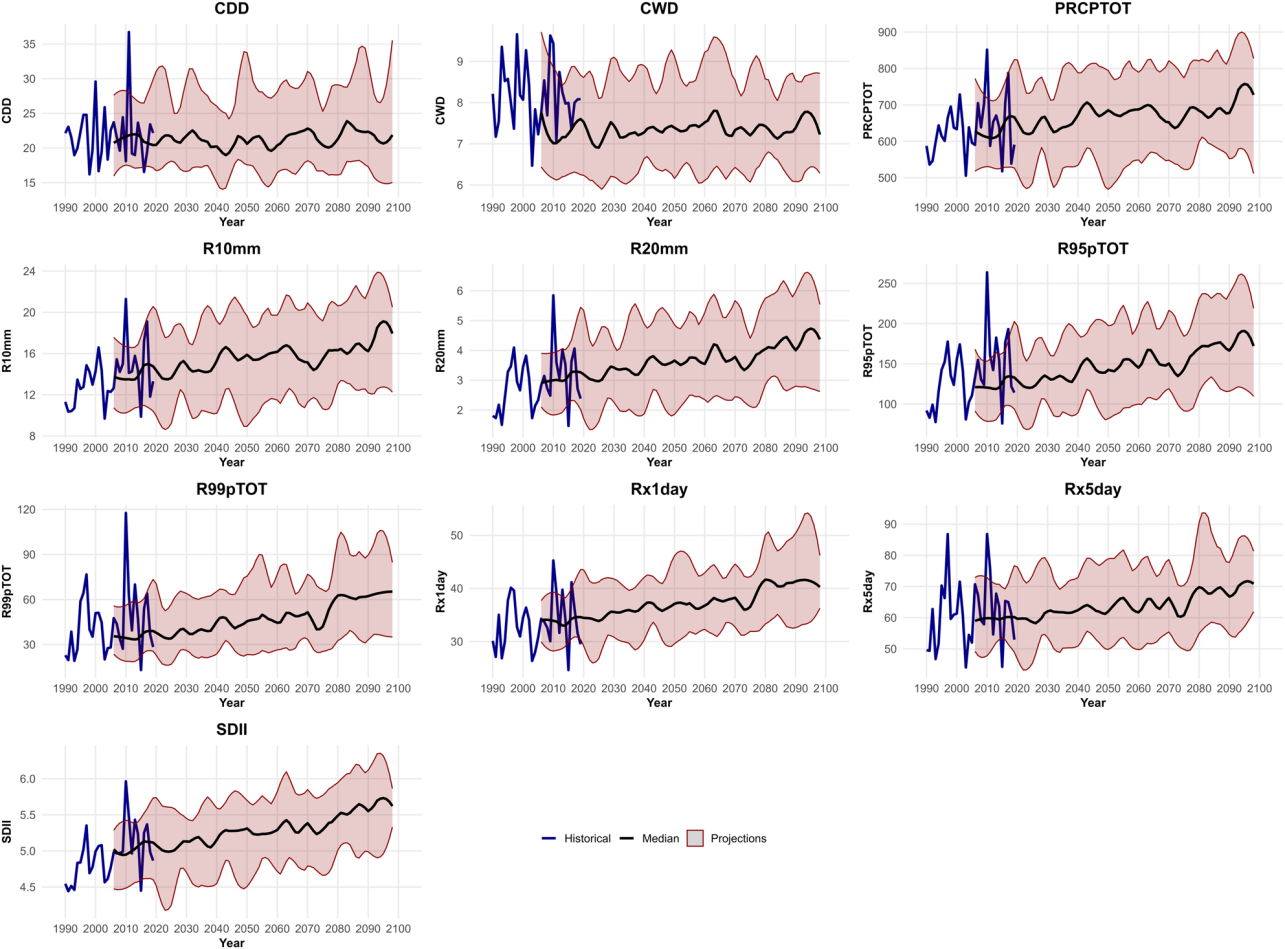
Time series of ETCCDI indicators over Poland, historical and projections.

The time series plots (Fig. 2) show the historical values and the projected future changes for each index until 2100.

#### Duration indices

*Consecutive Dry Days (CDD):* Historically, there is variability in the longest dry spells. The future projections for CDD show a less clear trend compared to intensity and frequency indices. The median line remains relatively flat or shows a very slight decrease towards the end of the century for some models, while the uncertainty band is quite large. Some models project slight increases, others slight decreases. This suggests considerable uncertainty regarding changes in the length of the longest dry spells, with no strong consensus signal for a significant increase or decrease across the model ensemble for the region as a whole.

*Consecutive Wet Days (CWD):* Historically, the length of the longest wet spells varies. Similar to CDD, the future projections for CWD do not show a strong, consistent trend across the models. The median line is relatively flat, perhaps with a very slight upward tendency. The uncertainty range is substantial. This indicates low confidence in significant changes to the length of the longest wet spells.

#### Total precipitation and general intensity

*Total Wet-day Precipitation (PRCPTOT)***:** Historically, the total annual precipitation on wet days shows considerable year-to-year variability. Looking towards the far future, the median projection across the models indicates a clear increasing trend during the twenty-first century. The average annual precipitation is projected to rise steadily, potentially increasing by 100–150 mm by the end of the century compared to the late 20th or early 21st-century baseline. The spread between the models also increases over time, signifying growing uncertainty in the exact magnitude of the increase, but the overall upward trend is consistent across the ensemble.

*Simple Daily Intensity Index (SDII)***:** This index measures the average precipitation amount on a wet day (total precipitation divided by the number of wet days). Similar to total rainfall, historical data shows fluctuations. The future projections consistently show an increasing trend in the median SDII. This suggests that rainfall is projected to have more intensity on average on rainy days. The increase appears relatively steady throughout the century, with the median value rising by approximately 0.5 to 1.0 mm/day by 2100. The uncertainty range also widens, particularly in the second half of the century.

#### Extreme precipitation intensity indices

*Maximum 1-day Precipitation (Rx1day):* The historical data shows significant peaks, indicating years with very intense single-day rainfall events. The future projections show a clear upward trend in the median maximum 1-day precipitation. This suggests that the intensity of the heaviest single-day rainfall events is expected to increase. By the end of the century, the median projection suggests these events could be roughly 5–10 mm higher than the typical values seen historically. The model spread is considerable and increases over time, highlighting uncertainty about how much more intense these single-day events will become.

*Maximum 5-day Precipitation (Rx5day):* Similar to Rx1day, historical data shows variability. The future projections indicate a consistent increasing trend in the median Rx5day values. This points towards more intense multi-day rainfall events. The projected increase by 2100 is substantial, potentially reaching ~ 10–20 mm higher than historical averages for the median projection. Again, the uncertainty range widens significantly towards the end of the century.

*Precipitation Fraction from Very Wet Days (R95pTOT):* Historically, this fraction varies. The future projections show a marked increasing trend in the median R95pTOT. This indicates that a larger share of the total annual rainfall is expected to come from these very heavy rainfall events in the future. The increase is projected to be quite significant by 2100.

*Precipitation Fraction from Extremely Wet Days (R99pTOT):* The historical data shows high variability, with some years having a significant fraction of rain from just a few extreme events. The future projections show a strong increasing trend in the median R99pTOT, even more pronounced than R95pTOT. This strongly suggests that the contribution of the most extreme rainfall events to the total annual precipitation will increase significantly under the RCP8.5 scenario. The uncertainty range is very wide, reflecting the inherent variability and model differences in simulating such rare, extreme events.

#### Frequency of heavy precipitation indices

*Number of Heavy Precipitation Days (R10mm):* Historically, the number of such days fluctuates. The future projections show a clear increasing trend in the median number of R10mm days. This indicates that days with moderate to heavy rainfall are likely to occur more often. The estimated rise by 2100 could result in several additional such days annually compared to the historical average.

*Number of Very Heavy Precipitation Days (R20mm)***:** The future projections show an increasing trend in the median number of R20mm days, suggesting very heavy rainfall days will also become more common. The relative increase might be larger than R10mm, although the absolute number of such days remains lower.

### Comparison of model projections and historical data

**Fig. 3:**
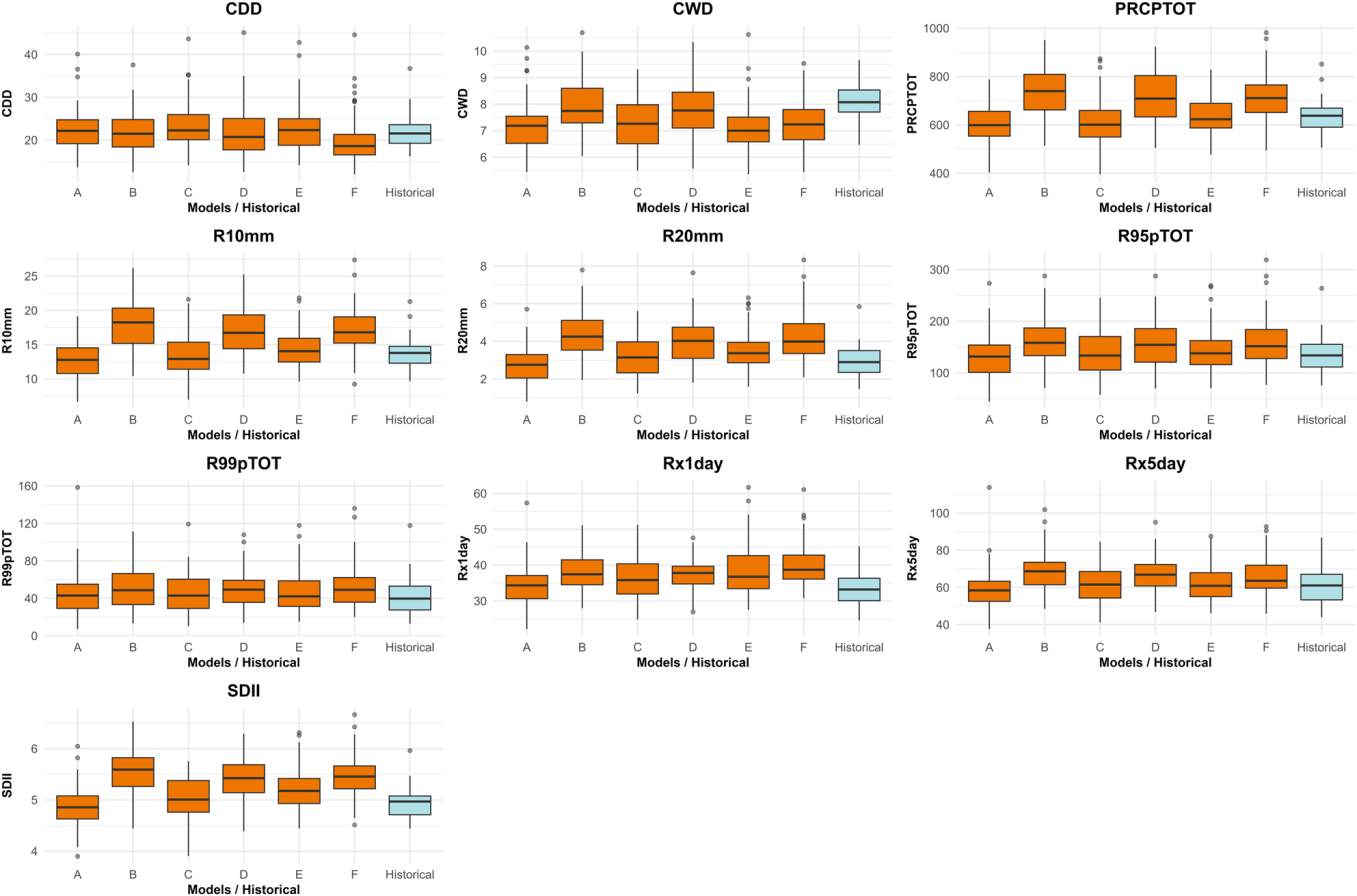
Comparison of model projections and historical data using box plots.

The box plots (Fig. 3) compare the overall distribution (median, interquartile range, typical range, and outliers) of each index as simulated by the individual climate models (labeled A through F) for the future period (2006–2100) against the distribution observed in the historical data (labeled ‘Historical’, 1990–2019).

For most indices related to precipitation intensity and frequency (PRCPTOT, SDII, Rx1day, Rx5day, R95pTOT, R99pTOT, R10mm, R20mm), the box plots show that the projected future distributions from all or most models (A-F) are shifted upwards compared to the historical distribution, considering that historical box plots contains 30 years but the projections cover 94 years. This visually confirms the increasing trends seen in the time series plots. The median values projected by the models for the future period are generally higher than the historical median.

There is noticeable variability among the different models (A-F). For instance, for PRCPTOT, models B, D, and F tend to project higher total precipitation compared to models A, C, and E. Similar variations exist for other indices, reflecting the inherent uncertainty in climate projections stemming from different model physics and parameterizations. Some models consistently project higher extremes than others across multiple indices (e.g., Model B often shows higher values for intensity and frequency indices).

While the future median values are generally higher for intensity/frequency indices, the projected ranges often overlap with the historical range, especially for the lower end of the projections. However, the upper ends of the projected ranges, including outliers, often extend well beyond the historically observed maximums, particularly for indices like Rx1day, Rx5day, and R99pTOT. This highlights the potential for future events to exceed the intensity or frequency of anything experienced in the historical record.

For CDD, the box plots show less consistent shifts. Some models (like F) project a lower median CDD compared to historical, while others are similar or slightly higher. The overall range projected by the models largely overlaps with the historical range, reinforcing the uncertainty about future changes in long dry spells. For CWD, the projected medians are mostly similar to or slightly higher than the historical median, but again, there’s considerable overlap in the ranges and variability between models, indicating no strong signal of change.

Many models project outlier events that are significantly more extreme than the typical range for intensity and frequency indices, often exceeding historical outliers. This emphasizes the increased risk of unprecedented extreme rainfall events in the future under this scenario.

In summary, the results consistently point towards a future with generally higher total precipitation, driven by more frequent heavy rainfall days and, more significantly, by an increase in rainfall intensity, especially during the most extreme events (single-day, multi-day, and percentile-based extremes). A larger fraction of the total rainfall is expected to fall during these intense episodes. Changes in the duration of wet and dry spells are less certain, with considerable variability among models and less clear overall trends compared to the intensity and frequency metrics. The comparison across models highlights the inherent uncertainties, particularly in the magnitude of change for the most extreme indices. Still, the overall change direction towards more intense and frequent heavy rainfall is a robust signal across the model ensemble for this high-emission scenario.

### Spatial distribution of ETCCDI indicators

**Fig. 4:**
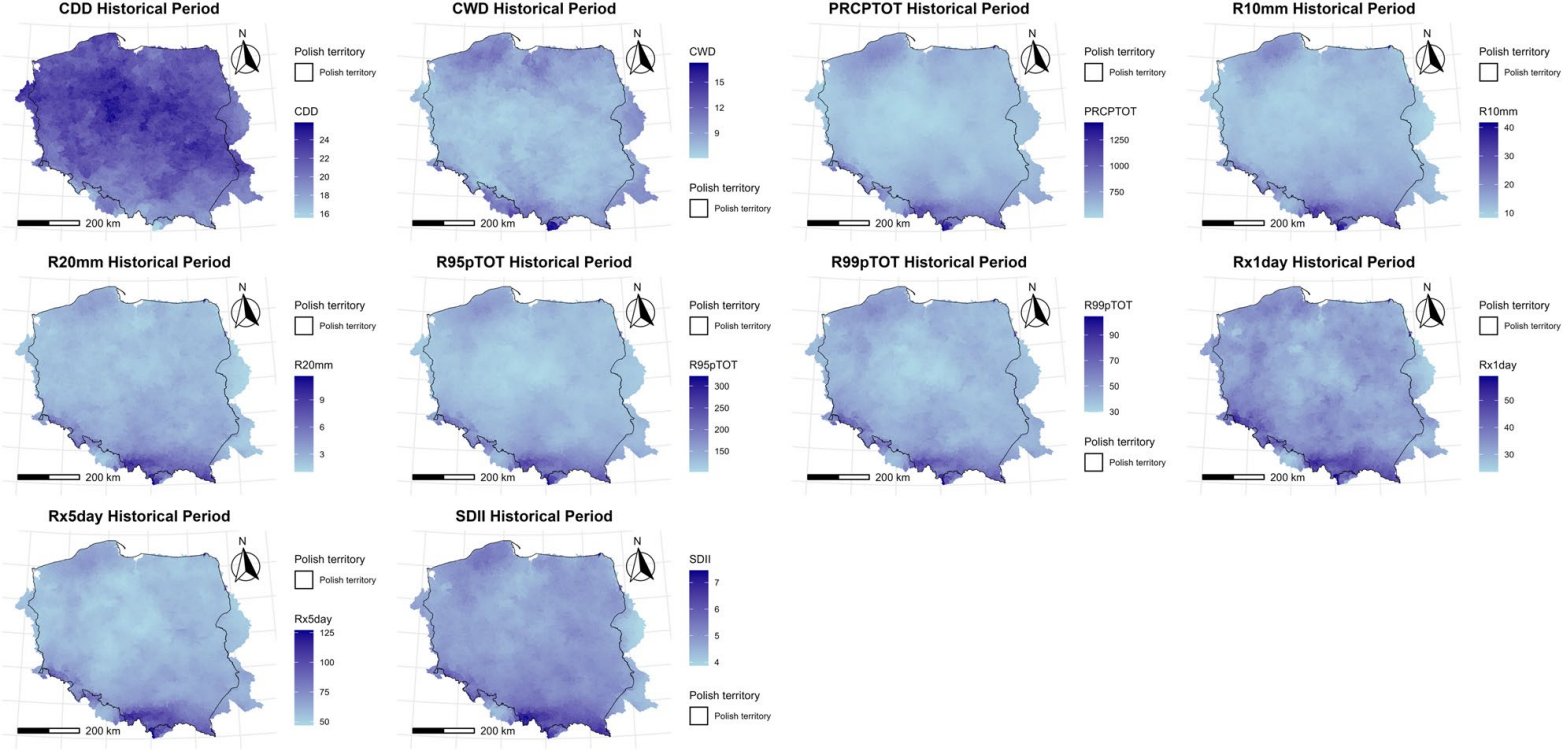
Spatial distribution of ETCCDI indicators over the study area.

This section examines the spatial distribution of indicators for historical and projected data (Fig. 4).

#### Historical (1990–2019) spatial distribution

The map of CDD shows that most areas in Poland had between 18 and 24 consecutive dry days. The lowest values are in the south, especially in the mountainous regions, while the highest values are more concentrated in the central parts. This indicates that longer dry spells are more common in eastern and central Poland, while the south sees slightly more frequent rainfall.

According to the CWD plot, the range of CWD mostly falls between 9 and 15 days. The distribution is fairly even across the country, though slightly higher values are observed in the southern (mountainous) areas. These regions experience more persistent wet periods, likely due to orographic rainfall.

Based on PRCPTOT, the majority of Poland receives less than 750 mm of rain per year. Higher precipitation is found in the south due to elevation (e.g., the Carpathian and Sudeten Mountains). The north and central parts receive less total rainfall. Overall, precipitation is more intense and frequent in the country’s southern parts.

R10mm (days with over 10 mm of rain) shows a national average of around 20 to 25 days per year. The southern areas again stand out, with more heavy rainfall days, particularly near the mountainous borders. R20mm (over 20 mm of rain per day) is much less frequent, with most places showing between 3 and 9 such days annually. The pattern is similar to R10mm but more localized, with the southern region and some parts in the north receiving the highest values. This confirms that intense rainfall is more common in elevated areas, where orographic lift enhances precipitation rates.

R95pTOT (total rainfall from days above the 95th percentile) ranges between ~ 150 mm and ~ 300 mm. R99pTOT (for days above the 99th percentile) shows values between ~ 30 mm and ~ 90 mm. Both indicators show higher values in the south and southwest, meaning these areas experience more extreme rainfall events. These heavy events are likely to contribute significantly to flood risk in those regions.

Rx1day and Rx5day display the most rain received in a single day or in a 5-day period, respectively. Rx1day values range mostly from 30 to 55 mm, while Rx5day shows 50 mm to over 125 mm. Again, higher values appear in southern and southeastern regions. The maps confirm that these areas are wetter overall and experience more intense rainfall over short periods, increasing the likelihood of flash flooding and erosion.

SDII is the ratio of total rainfall to the number of wet days, showing how intense rainfall is on average. Most of Poland lies between 5 and 7 mm/day. Higher values are once again seen in southern Poland. This suggests that when it rains there, it tends to rain more heavily compared to other regions.

The maps (Fig. 4) show that southern Poland—especially areas near the Carpathian and Sudeten Mountains—consistently records higher values for all precipitation indicators. These include total rainfall, frequency of heavy and extreme rain events, and rainfall intensity. This pattern is likely due to orographic effects and local climatic conditions. In contrast, central Poland experiences fewer extreme events, lower total rainfall, and longer dry periods (as seen in the CDD map). These regions may be more prone to droughts, particularly in agriculturally intensive areas where water availability is essential.

#### Spatial distribution of projections

**Fig. 5:**
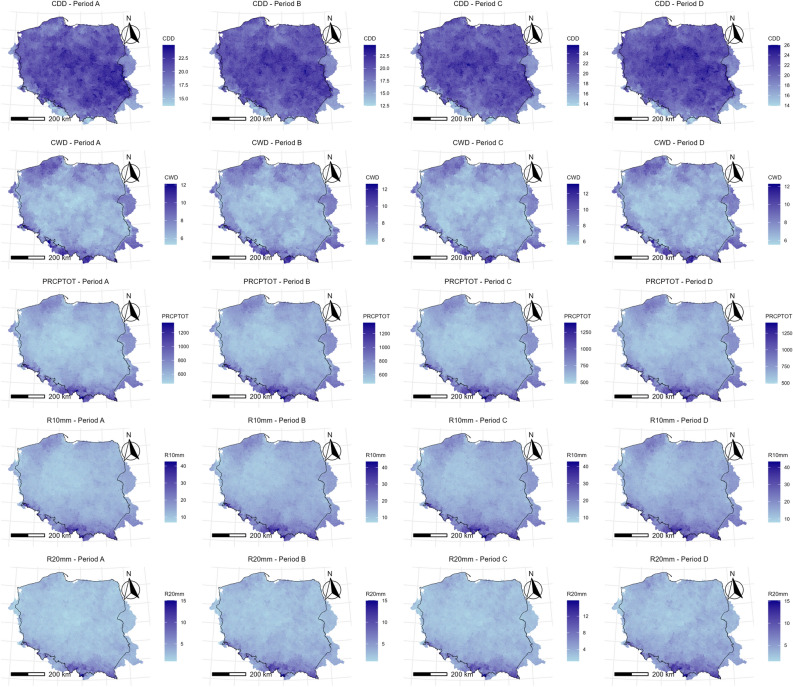
Spatial distributions of CDD, CWD, PRCPTOT, R10mm, R20mm for 2021–2040 (Period A), 2041–2060 (Period B), 2061–2080 (Period C), and 2081–2100 (Period D).

**Fig. 6:**
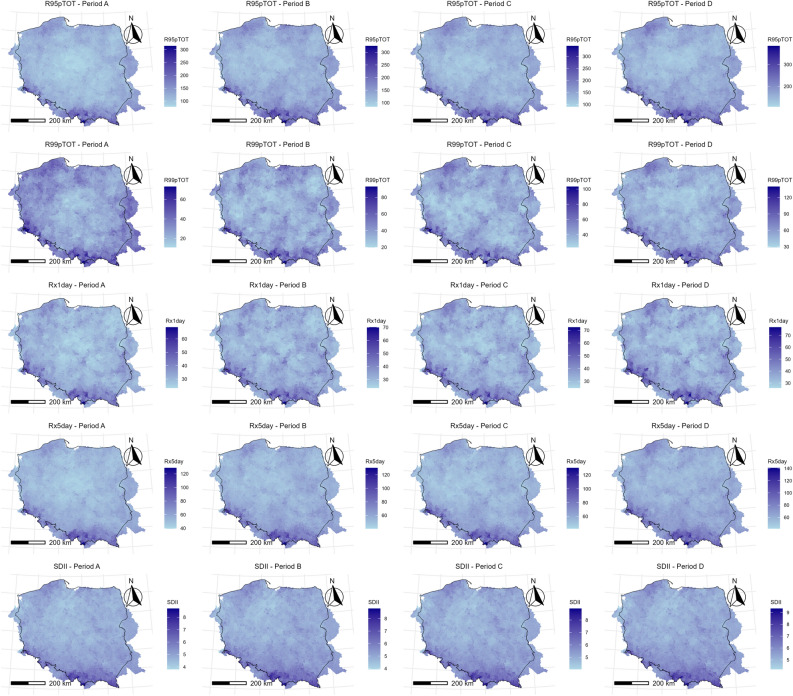
Spatial distributions of R95pTOT, R99pTOT, Rx1day, Rx5day, SDII for 2021–2040 (Period A), 2041–2060 (Period B), 2061–2080 (Period C), and 2081–2100 (Period D).

This section examines the projected spatial patterns of change for selected ETCCDI precipitation indices across the study area (Figs. 5 and 6). The analysis is based on the ensemble median of the six climate models under the RCP8.5 scenario, averaged over four consecutive 20-year periods: 2021–2040 (Period A), 2041–2060 (Period B), 2061–2080 (Period C), and 2081–2100 (Period D).

Consecutive Dry Days (CDD): Across all four periods, the spatial pattern remains fairly consistent, with most regions showing values between roughly 18 and 24 days. There is a slight tendency for longer dry spells in the central and eastern parts of Poland compared to the southern mountainous regions and the northwest, where values are slightly lower. Comparing the periods from A to D, there are no remarkable shifts in the spatial pattern or intensity. The changes over time appear minimal and lack a clear geographical structure, which aligns with the previous analysis showing no significant trend for CDD for the region as a whole.

Consecutive Wet Days (CWD): The projected maximum length of wet spells shows a clearer spatial pattern than CDD. The longest wet spells (values > 8 days) are consistently projected for the southern mountainous regions (Carpathians and Sudetes) and, to some extent, the northeastern parts of the study area. The central lowlands consistently show shorter maximum wet spells (around 6–7 days). Similar to CDD, the changes across the four future periods (A to D) are very slight. There might be a slight intensification of CWD in the south by Period D, but the overall spatial pattern remains largely stable, consistent with the lack of a strong, significant trend found in the temporal analysis.

Total Wet-day Precipitation (PRCPTOT): The spatial distribution of total annual precipitation clearly reflects the topography. The highest amounts (over 800–900 mm) are consistently projected for the southern mountain ranges. The lowest amounts (around 500–600 mm) are projected for the central Polish lowlands. Moving from Period A to Period D, a clear and widespread increase in total precipitation is evident across the entire study area. While the general south-to-center gradient persists, the increase appears substantial everywhere. By Period D (2081–2100), large parts of the country are projected to receive over 700 mm annually, with the southern regions exceeding 900–1000 mm according to the ensemble median.

Number of Heavy Precipitation Days (R10mm): The frequency of days with rainfall exceeding 10 mm also shows a distinct spatial pattern, strongly related to total precipitation. The highest number of R10mm days (> 20 days/year) occurs in the southern mountains. Central Poland typically shows the lowest frequency (around 12–15 days/year). Similar to PRCPTOT, there is a clear and consistent increase in the number of R10mm days across the entire study area from Period A to Period D. The frequency increases in all regions, maintaining the south-to-center gradient but with noticeable rises even in the drier central areas. By Period D, the southern regions are projected to experience over 25 such days annually, while central regions may see around 16–18 days.

Number of Very Heavy Precipitation Days (R20mm): The spatial pattern for days with rainfall exceeding 20 mm mirrors that of R10mm but with lower absolute frequencies. The highest frequency (4–6 days/year initially, increasing to 6–8 + days/year) is concentrated in the southern mountains. Central and northern regions initially show very low frequencies (1–2 days/year). A pronounced increase in R20mm days is projected across all regions from Period A to D. While the absolute highest frequencies remain in the south, the relative increase appears substantial throughout the study area. By Period D, even the central and northern lowlands are projected to experience a higher frequency (around 3–4 days/year) of these very heavy rainfall events compared to the early century.

Precipitation Fraction from Very Wet Days (R95pTOT): Spatially, the highest contributions tend to occur in the southern mountains and parts of the north/northwest. Central Poland generally shows slightly lower contributions. From Period A to Period D, there is a noticeable increase in this fraction across the entire study area. The spatial gradient remains, but the overall contribution increases throughout the region, consistent with the significant increasing trend found earlier.

Precipitation Fraction from Extremely Wet Days (R99pTOT): The spatial pattern is less distinct than R95pTOT, but slightly higher contributions are often seen in the south and northwest. Central areas show lower contributions. A clear and strong increase is projected across all regions from Period A to Period D. By the end of the century (Period D), large portions of the study area show contributions exceeding 10–12%, indicating a significantly larger role for the most extreme events in the total rainfall budget. This aligns with the strong significant increasing trend identified previously.

Maximum 1-day Precipitation (Rx1day): The highest single-day rainfall amount shows a clear spatial pattern, with the most intense events (> 40–45 mm) projected for the southern mountainous regions. Central and northern areas generally show lower Rx1day values (around 30–35 mm). A consistent increase in Rx1day intensity is projected across the entire study area from Period A to Period D. The values increase everywhere, with the southern mountains projected to experience Rx1day events exceeding 50 mm by Period D, while central and northern areas see increases towards 40 mm. This spatial pattern reflects the significant increasing trend found for Rx1day.

Maximum 5-day Precipitation (Rx5day): The pattern for the heaviest 5-day rainfall totals is very similar to Rx1day. The highest values (> 70–80 mm) are concentrated in the southern mountains, while central and northern areas show lower totals (50–60 mm). A substantial and widespread increase in Rx5day is projected from Period A to Period D. By the end of the century, the southern regions are projected to see maximum 5-day totals exceeding 90–100 mm, while central and northern areas increase towards 70–80 mm. This confirms the significant increasing trend previously identified.

Simple Daily Intensity Index (SDII): This index represents the average rainfall amount on a wet day. Spatially, the highest intensities (> 5.5–6.0 mm/day) are projected for the southern mountains. Central Poland shows lower average intensities (around 4.5–5.0 mm/day). A clear increasing trend in SDII is visible across the entire study area from Period A to Period D. Average wet-day intensity increases everywhere, with the southern regions potentially exceeding 6.5 mm/day and central areas increasing towards 5.5 mm/day by the end of the century. This spatial visualization reinforces the significant increasing trend found for SDII both historically and in projections.

In summary, the spatial analysis for all examined indicators reveals consistent patterns. Indices related to precipitation intensity (SDII, Rx1day, Rx5day) and the contribution of extremes (R95pTOT, R99pTOT) show the highest values predominantly in the southern mountainous regions, mirroring the patterns seen for total precipitation (PRCPTOT) and frequency of heavy events (R10mm, R20mm). Crucially, all these intensity, frequency, and contribution indices exhibit clear, widespread increases across the entire study area throughout the twenty-first century under the RCP8.5 scenario. While existing geographical gradients are expected to persist, the magnitude of extreme precipitation is projected to increase substantially everywhere. In contrast, indices related to the duration of dry (CDD) and wet (CWD) spells show much weaker spatial gradients and minimal, inconsistent changes over time.

## Discussion

Our analysis of extreme precipitation over the Vistula and Oder/Odra basins indicates that moderate increases in heavy precipitation have already occurred in recent decades, and much larger changes are projected by the end of the twenty-first century. These findings are broadly consistent with observed trends and model projections reported in the literature for Central Europe and other regions^22^. In the historical period (1990–2019), we found slight upward trends in indices like heavy precipitation days (R10mm) and very heavy days (R20mm), while the most extreme intensity indices (annual maximum 1-day and 5-day precipitation, Rx1day and Rx5day) and total wet-day precipitation (PRCPTOT) showed no statistically significant change. This aligns with station-based studies in Poland and Europe that report mixed but generally increasing tendencies in intense rainfall over recent decades ^33–36^.

For instance, a comparison of 1961–1990 vs 1991–2015 in Poland showed that most measures of intense precipitation (e.g. days ≥ 10 mm or ≥ 20 mm, multi-day totals) were higher in the recent period, especially in summer, although many trends were weak or not significant due to high variability​. This reflects the broader European pattern noted by^37^ that intense precipitation has typically increased across Europe in the late twentieth century, albeit not uniformly everywhere^36^.

Our results emphasize that the climate change signal in heavy precipitation is emerging in Central Europe, even if detection at individual locations remains challenging over short records. Looking forward, the EURO-CORDEX model projections under RCP8.5 (2006–2100) point to robust and significant increases in all examined precipitation indices related to wet extremes. We found that by the late twenty-first century, annual precipitation totals (PRCPTOT) in the Vistula and Oder basins could rise markedly, and the frequency (R10mm, R20mm) and intensity (Rx1day, Rx5day) of heavy rain events are projected to increase substantially. Numerous studies using regional and global climate models strongly support this future intensification of precipitation extremes. For example, high-resolution regional model ensembles for Europe project large increases in heavy precipitation intensity—on the order of + 20–35% in Central and Eastern Europe by 2071–2100 under a high-emission scenario^38^. Even at the global scale, climate models consistently show that indices like Rx1day and Rx5day will increase in virtually all regions as the climate warms​^36,39,40^.

Our findings agree with these projections: in Central Poland, a recent CMIP6-based study likewise expects mean annual precipitation to increase by a few percent (up to ~ 7% by century’s end) along with a rise in the number of days exceeding 10 mm, 20 mm, and 30 mm of rain^41^. However, the direction of change is the same, but the magnitude is lower. The projected increases in very wet (R95pTOT) and extremely wet (R99pTOT) precipitation contributions in our study indicate that a greater share of yearly rainfall will come from intense events in the future. This outcome is also reported in other regions and models—heavier downpours are expected to contribute disproportionately more to annual rainfall under continued warming^36,41,42^.

Overall, the direction and magnitude of projected changes in these ETCCDI indices over our study area are in line with previous EURO-CORDEX and CMIP5/6 studies, adding confidence that our regional results are representative of broader climate change patterns. It is worth noting that the patterns of change in precipitation extremes are not uniform across Europe. Our focus basins in Central Europe are projected to experience significant increases in heavy precipitation in all seasons. This corresponds with findings that northern and central parts of Europe will likely see the strongest uptick in extreme precipitation intensities. In winter, for instance, model projections under RCP8.5 show the largest increases (up to ~ 35%) in heavy precipitation occurring over Central and Eastern Europe^38^. In contrast, some regions of Southern Europe may follow a different trajectory. Mean precipitation in the Mediterranean is expected to decrease with warming, yet paradoxically extreme rainfall events in those areas could become more intense or frequent. Studies have noted that even where average summer rainfall declines (e.g. Iberian Peninsula), the most extreme rain events can still intensify due to higher atmospheric moisture availability^38,43^. For example, heavy summer precipitation could decrease by ~ 25% in parts of Spain under RCP8.5, but at the same time, winter extremes increase, and overall, the Mediterranean region still shows increases in short-duration downpour intensity^38^. Sillmann et al.^44^ similarly found that southern Europe’s drying does not preclude increases in heavy precipitation—a robust result of climate models is that the frequency of very intense rain events rises even in some currently dry regions​.

Therefore, while our study basins are firmly in the “wetter” part of Europe’s climate change gradient (with increasing totals and extremes), the comparison with other regions underscores that climate change can simultaneously cause both more intense rainfall extremes and longer dry spells, depending on the location and season​. This nuanced pattern has been observed in other national studies as well—for instance, increasing heavy rain days in northwestern Poland coupled with more summer dry days in the south^36,42,45^—highlighting the complex spatial nature of precipitation change under global warming. Our use of the ETCCDI indices enables direct comparison with many similar studies that have analyzed extreme precipitation. Numerous authors have applied these standardized indices to both observational data and climate model outputs across Europe. Overall, our findings reinforce the consensus that climate change is likely to intensify the hydrological cycle and wet extremes. Prior studies with EURO-CORDEX have reported analogous increases in extreme precipitation indices for various European regions (e.g. the Alps and Iberia), especially under high-end scenarios, after accounting for model biases. Likewise, global multi-model studies (CMIP5/6) of ETCCDI metrics conclude that indices such as R95pTOT, Rx5day, and R20mm will increase in virtually all land areas by the late century under RCP8.5​^42,45^.

This consistency across independent studies and regions strengthens confidence in our projections for the Vistula and Oder basins. Small differences in magnitude can arise due to regional factors—for example, our study projects a slightly larger increase in heavy rainfall frequency than some pan-European averages, which could be related to local topography (e.g. orographic enhancement in the southern basin areas) or model ensemble specifics. However, the direction of change (increasing heavy precipitation and intensity) is clearly supported by the literature. In summary, our results are part of a growing body of evidence, from Poland, Central Europe, and globally, that extreme precipitation indices are on the rise in response to anthropogenic warming^38,39,41,42,44^.​

These projected changes in precipitation extremes have important implications for flood risk and water management in the region. Heavier and more frequent downpours will elevate the risk of pluvial and fluvial flooding in the Vistula and Oder catchments. Historical flood records in Europe do not yet show clear upward trends, partly because detecting trends in rare floods is difficult, given the large natural variability​^46^. In fact, despite observed increases in heavy rainfall, long-term discharge series in Central Europe have not conclusively indicated more frequent floods to date​^46,47^.

This suggests that adaptation measures should be proactive: our future projections imply that the absence of a strong past trend may be due to the short observational period or confounding factors, and it should not engender complacency. Model-based studies warn that extreme river flood events are likely to become much more frequent under continued high GHG emissions. For example, what is currently deemed a 100-year flood in Europe could occur roughly twice as often by mid- to late-century under scenarios like RCP8.5^48^.

In our basins, increased Rx5day values point to a higher probability of severe multi-day rainfall episodes, often the drivers of large-scale river floods. This calls for strengthened flood risk management strategies. Potential adaptation actions include upgrading flood defenses (levees, retention basins) to accommodate higher flood peaks, improving urban drainage systems to handle intense cloudbursts, and preserving floodplains to buffer high flows. Additionally, water resource management will need to adapt to more variability—periods of heavy rainfall may provide more water, but if a greater share comes in extreme events, capturing and storing that water without damage becomes a challenge. Indeed, an “extreme abundance” of water in short bursts can lead to soil erosion, flash floods, and infrastructure stress^36^. Conversely, longer dry spells (if they coincide with the changes) can strain the water supply and agriculture​^36^.

The findings from this study underscore the urgency for policymakers and planners to integrate the increasing likelihood of extreme precipitation into their risk assessments and adaptation plans. Enhancing the resilience of communities in the Vistula and Oder basins—for example, by updating design standards for drainage, roads, and dams to account for heavier rain, and by implementing early warning systems for extreme weather—will be crucial in mitigating the impacts of these projected changes. Our results provide a scientific basis to inform such adaptation efforts, and they echo the conclusions of other studies that highlight climate change’s profound impact on the water cycle and flood hazard across Europe​^36^. Each region will have its own specific vulnerabilities, but a common message emerges: the increase in extreme precipitation under climate change is expected to continue, and societies must prepare for more frequent heavy rainfall events and their consequences. By comparing our regional findings with the broader literature, we gain confidence in this outcome and emphasize the need for coordinated adaptation strategies to manage the coming changes in flood risk and water resources.

While the ensemble approach captures plausible scenarios, significant inter-model spread persists, particularly for extreme precipitation. To reduce this uncertainty, future research could apply the Emergent Constraint (EC) framework. This method leverages physical relationships between current observations and future projections to constrain the probable range of outcomes more effectively than simple multi-model averages.This approach has effectively narrowed uncertainty in climate sensitivity^49^ and global precipitation^50^. Recent studies have utilized the framework to refine projections of global water availability^51^, Northern Hemisphere snowmelt^52^, land greening impacts^53^, and Asian climate trends^54^. Adopting this framework for the Baltic Sea region could similarly reduce uncertainties in extreme precipitation projections, supporting robust adaptation planning.

## Conclusion

This study analyzed historical trends and future projections of extreme precipitation in the Vistula and Oder river basins within the Baltic Sea region, utilizing the ETCCDI indices and bias-corrected EURO-CORDEX climate model simulations under the RCP8.5 scenario.

### Main conclusions

- *Historical vs. Future Trends:* While historical data (1990–2019) showed only slight, often non-significant upward trends in heavy precipitation days (R10mm) and intensity (SDII) due to natural variability, future projections (2006–2100) under the high-emission scenario demonstrate robust, statistically significant increases across nearly all wet extreme indices.
- *Intensification of Extremes:* There is a clear signal for increased frequency and intensity of heavy rainfall. Specifically, the median projection for the maximum 5-day precipitation (Rx5day), a critical driver for riverine flooding, is projected to increase by approximately 10–20 mm by 2100 compared to historical averages. Similarly, total annual precipitation (PRCPTOT) is projected to rise by 100–150 mm by the end of the century.
- *Contribution of extremes*: The proportion of total rainfall derived from the most extreme events (R95pTOT and R99pTOT) is projected to rise significantly. This indicates a shift in the regional water budget where a larger share of annual water availability will occur during short, intense episodes.
- *Spatial heterogeneity:* The southern mountainous regions (Carpathians and Sudetes) historically exhibited the highest extreme precipitation values and are projected to experience the most pronounced absolute increases (e.g., Rx5day exceeding 90–100 mm). However, significant relative increases are projected broadly across the entire study area, including the central lowlands.
- *Uncertainty in duration:* Unlike intensity metrics, duration indices for consecutive dry (CDD) and wet (CWD) days showed high inter-model variability and no clear trend, suggesting greater uncertainty regarding future spell lengths.

### Recommendations for adaptation and future research

- *Engineering Design Standards:* Given the projected increases in Rx1day (5–10 mm) and Rx5day (10–20 mm), current hydraulic design standards (e.g., Intensity–Duration–Frequency curves) should be revisited. Infrastructure such as urban drainage systems and dams must be upgraded to accommodate these higher peak loads to prevent structural failure.
- Flood risk management**:** The significant rise in R95pTOT and R99pTOT implies a higher risk of flash floods and pluvial flooding. Flood defense strategies should prioritize expanding retention basins and preserving floodplains, particularly in the southern mountainous catchments where changes are most severe.
- *Future research directions:* Future studies should focus on reducing the uncertainty observed in dry/wet spell duration (CDD/CWD). Additionally, applying the Emergent Constraint (EC) method could help narrow the uncertainty range in future precipitation projections by utilizing observational constraints to weight climate models.

## Data Availability

The datasets used and/or analyzed during the current study are available from the corresponding author on reasonable request.

## References

[1] Abro, M. I., Zhu, D., Elahi, E., Majidano, A. A. & Solangi, B. K. Hydrological simulation using multi-sources precipitation estimates in the Huaihe River Basin. *Arab. J. Geosci.* **14**, 1912 (2021).

[2] Delavar, M., Morid, S., Zaghiyan, M. R., & Eini, M. R. Climate change and intensified irrigation as drivers of water imbalance in a large dry basin with two vanishing lakes in Iran. In *Environment, Development and Sustainability* 1–31 (2025)

[3] Rojpratak, S. & Supharatid, S. Regional extreme precipitation index: Evaluations and projections from the multi-model ensemble CMIP5 over Thailand. *Weather Clim. Extremes* **37**, 100475 (2022).

[4] Vinod, D. & Mahesha, A. Large-scale atmospheric teleconnections and spatiotemporal variability of extreme rainfall indices across India. *J. Hydrol.* **628**, 130584. 10.1016/j.jhydrol.2023.130584 (2024).

[5] Yin, H. & Sun, Y. Characteristics of extreme temperature and precipitation in China in 2017 based on ETCCDI indices. *Adv. Clim. Change Res.* **9**, 218–226. 10.1016/j.accre.2019.01.001 (2018).

[6] Al-Sakkaf, A. S. et al. Assessing exposure to climate extremes over the Arabian Peninsula using ERA5 reanalysis data: Spatial distribution and temporal trends. *Atmos. Res.* **300**, 107224 (2024).

[7] Bastola, S., Cho, J., Kam, J. & Jung, Y. Assessing the influence of climate change on multiple climate indices in Nepal using CMIP6 global climate models. *Atmos. Res.* **311**, 107720. 10.1016/j.atmosres.2024.107720 (2024).

[8] Eini, M. R., Najminejad, F. & Piniewski, M. Direct and indirect simulating and projecting hydrological drought using a supervised machine learning method. *Sci. Total Environ.* **898**, 165523 (2023). 37454850 10.1016/j.scitotenv.2023.165523

[9] Eini, M. R., Salmani, H. & Piniewski, M. Comparison of process-based and statistical approaches for simulation and projections of rainfed crop yields. *Agric. Water Manag.* **277**, 108107 (2023).

[10] Panda, D. K., Panigrahi, P., Mohanty, S., Mohanty, R. K. & Sethi, R. R. The 20th century transitions in basic and extreme monsoon rainfall indices in India: Comparison of the ETCCDI indices. *Atmos. Res.* **181**, 220–235. 10.1016/j.atmosres.2016.07.002 (2016).

[11] Walsh, J. E. et al. Extreme weather and climate events in northern areas: A review. *Earth-Sci. Rev.* **209**, 103324 (2020).

[12] Knutzen, F. et al. Impacts on and damage to European forests from the 2018–2022 heat and drought events. *Nat. Hazards Earth Syst. Sci.* **25**, 77–117 (2025).

[13] Piniewski, M., Eini, M. R., Chattopadhyay, S., Okruszko, T. & Kundzewicz, Z. W. Is there a coherence in observed and projected changes in riverine low flow indices across Central Europe?. *Earth-Sci. Rev.* **233**, 104187 (2022).

[14] Szyga-Pluta, K., Tomczyk, A. M., Piniewski, M. & Eini, M. R. Past and future changes in the start, end, and duration of the growing season in Poland. *Acta Geophys.* **71**, 3041–3055 (2023).

[15] Tomczyk, A. M., Piniewski, M. & Eini, M. R. Past and future changes in maximum air temperature and cold days in winter in Poland. *Acta Geophys.*10.1007/s11600-025-01538-0 (2025).

[16] Tomczyk, A. M., Piniewski, M., Eini, M. R. & Bednorz, E. Projections of changes in maximum air temperature and hot days in Poland. *Int. J. Climatol.*10.1002/joc.7530 (2022).

[17] Eini, M. R., Rahmati, A., Salmani, H., Brocca, L. & Piniewski, M. Detecting characteristics of extreme precipitation events using regional and satellite-based precipitation gridded datasets over a region in Central Europe. *Sci. Total Environ.* **852**, 158497. 10.1016/j.scitotenv.2022.158497 (2022). 36063945 10.1016/j.scitotenv.2022.158497

[18] Eini, M. R. et al. Detecting drought events over a region in Central Europe using a regional and two satellite-based precipitation datasets. *Agric. For. Meteorol.* **342**, 109733. 10.1016/j.agrformet.2023.109733 (2023).

[19] Hänsel, S., Ustrnul, Z., Łupikasza, E. & Skalak, P. Assessing seasonal drought variations and trends over Central Europe. *Adv. Water Resour.* **127**, 53–75 (2019).

[20] Hari, V., Rakovec, O., Markonis, Y., Hanel, M. & Kumar, R. Increased future occurrences of the exceptional 2018–2019 Central European drought under global warming. *Sci. Rep.* **10**, 12207 (2020). 32764540 10.1038/s41598-020-68872-9 PMC7413549

[21] Marcinkowski, P., Eini, M. R., Venegas‐Cordero, N., Jefimow, M. & Piniewski, M. Diverging projections of future droughts in high‐end climate scenarios for different potential evapotranspiration methods: A national‐scale assessment for Poland. *Int. J. Climatol.* **44**, 5902–5917 (2024).

[22] Kimutai, J., Vautard, R., Zachariah, M., Tolasz, R., Šustková, V., Cassou, C.. Skalák, P., Clarke, B., Haslinger, K., Vahlberg, M. Climate change and high exposure increased costs and disruption to lives and livelihoods from flooding associated with exceptionally heavy rainfall in Central Europe (2024).

[23] Meresa, H. K., Osuch, M. & Romanowicz, R. Hydro-meteorological drought projections into the 21-st century for selected Polish catchments. *Water* **8**, 206 (2016).

[24] Marcinkowski, P., Piniewski, M. & Jefimow, M. Assessment of projected climate change impact on agro-climatic indicators in Poland. *Int. J. Climatol.* **43**, 6003–6019 (2023).

[25] Piniewski, M., Szcześniak, M., Kardel, I., Chattopadhyay, S. & Berezowski, T. G2DC-PL+: A gridded 2 km daily climate dataset for the union of the Polish territory and the Vistula and Odra basins. *Earth Syst. Sci. Data* **13**, 1273–1288 (2021).

[26] Montes, C., Acharya, N., Hassan, S. Q. & Krupnik, T. J. Intense precipitation events during the monsoon season in Bangladesh as captured by satellite-based products. *J. Hydrometeorol.* **22**, 1405–1419 (2021).

[27] Zhang, X. et al. Indices for monitoring changes in extremes based on daily temperature and precipitation data. *Wiley Interdiscip. Rev. Clim. Change* **2**, 851–870 (2011).

[28] Lei, X. et al. Does non-stationarity of extreme precipitation exist in the Poyang Lake Basin of China?. *J. Hydrol. Reg. Stud.* **37**, 100920. 10.1016/j.ejrh.2021.100920 (2021).

[29] Gilbert, R. O. *Statistical Methods for Environmental Pollution Monitoring* (Wiley, New York, 1987).

[30] Gocic, M. & Trajkovic, S. Analysis of changes in meteorological variables using Mann-Kendall and Sen’s slope estimator statistical tests in Serbia. *Glob. Planet. Change* **100**, 172–182 (2013).

[31] Sen, P. K. Estimates of the regression coefficient based on Kendall’s tau. *J. Am. Stat. Assoc.* **63**, 1379–1389 (1968).

[32] Akpovi, B. A. et al. Hydrological appraisal using multi-source rainfall data in PDM model over the Qinhuai River basin in China. *Arab. J. Geosci.* **15**, 236 (2022).

[33] Łupikasza, E., & Małarzewski, Ł. Climate change in Poland: past, present, future. In *Climate change in Poland: Past, Present, Future* 349–373. (Springer, 2021).

[34] Łupikasza, E. B. & Małarzewski, Ł. Trends in the indices of precipitation phases under current warming in Poland, 1966–2020. *Adv. Clim. Change Res.* **14**, 97–115. 10.1016/j.accre.2022.11.012 (2023).

[35] Pińskwar, I. Complex changes of extreme precipitation in the warming climate of Poland. *Int. J. Climatol.* **42**, 817–833 (2022).

[36] Pińskwar, I., Choryński, A., Graczyk, D. & Kundzewicz, Z. W. Observed changes in extreme precipitation in Poland: 1991–2015 versus 1961–1990. *Theor. Appl. Climatol.* **135**, 773–787 (2019).

[37] Sackl-Sharif, S., Goldgruber, E., Ausserhofer, J., Gutounig, R., & Reimerth, G. Flows of water and information: Reconstructing online communication during the 2013 european floods in Austria. In *Social Media Use in Crisis and Risk Communication* 155–181. (Emerald Publishing Limited 2018).

[38] Jacob, D. et al. EURO-CORDEX: New high-resolution climate change projections for European impact research. *Reg. Environ. Change* **14**, 563–578 (2014).

[39] Hay, J. E., Easterling, D., Ebi, K. L., Kitoh, A. & Parry, M. Conclusion to the special issue: Observed and projected changes in weather and climate extremes. *Weather Clim. Extrem.* **11**, 103–105 (2016).

[40] Polade, S. D., Pierce, D. W., Cayan, D. R., Gershunov, A. & Dettinger, M. D. The key role of dry days in changing regional climate and precipitation regimes. *Sci. Rep.* **4**, 4364 (2014). 24621567 10.1038/srep04364 PMC3952143

[41] Ghazi, B., Przybylak, R. & Pospieszyńska, A. Projection of climate change impacts on extreme temperature and precipitation in Central Poland. *Sci. Rep.* **13**, 18772 (2023). 37907786 10.1038/s41598-023-46199-5 PMC10618218

[42] Rutkowska, A., Willems, P., Mendoza Paz, S. & Ziernicka-Wojtaszek, A. Changes in precipitation patterns in poland derived from projected downscaled future climate data from CMIP5 and CMIP6. *Int. J. Climatol.* **45**(7), e8822 (2025).

[43] Sassi, M. et al. Impact of climate change on European winter and summer flood losses. *Adv. Water Resour.* **129**, 165–177 (2019).

[44] Sillmann, J., Kharin, V. V., Zwiers, F. W., Zhang, X. & Bronaugh, D. Climate extremes indices in the CMIP5 multimodel ensemble: Part 2. Future climate projections. *J. Geophys. Res. Atmos.* **118**, 2473–2493 (2013).

[45] Kalbarczyk, R. & Kalbarczyk, E. Risk of natural hazards caused by extreme precipitation in Poland in 1951–2020. *Water* **16**, 1705 (2024).

[46] Kundzewicz, Z. W. et al. Differences in flood hazard projections in Europe–Their causes and consequences for decision making. *Hydrol. Sci. J.* **62**, 1–14 (2017).

[47] Venegas-Cordero, N., Kundzewicz, Z. W., Jamro, S. & Piniewski, M. Detection of trends in observed river floods in Poland. *J. Hydrol. Reg. Stud.* **41**, 101098. 10.1016/j.ejrh.2022.101098 (2022).

[48] Alfieri, L., Burek, P., Feyen, L. & Forzieri, G. Global warming increases the frequency of river floods in Europe. *Hydrol. Earth Syst. Sci.* **19**, 2247–2260 (2015).

[49] Hall, A., Cox, P., Huntingford, C. & Klein, S. Progressing emergent constraints on future climate change. *Nat. Clim. Change* **9**, 269–278 (2019).

[50] Shiogama, H., Watanabe, M., Kim, H. & Hirota, N. Emergent constraints on future precipitation changes. *Nature* **602**, 612–616 (2022). 35197617 10.1038/s41586-021-04310-8

[51] Wu, Y. et al. Larger increase in future global terrestrial water availability than projected by CMIP6 models. *Innov. Geosci.* **2**, 100097-100091-100097-100099 (2024).

[52] Chai, Y. et al. Constrained Earth system models show a stronger reduction in future Northern Hemisphere snowmelt water. *Nat. Clim. Change.*10.1038/s41558-025-02308-y (2025).

[53] Chai, Y. et al. Underestimating global land greening: Future vegetation changes and their impacts on terrestrial water loss. *One Earth*10.1016/j.oneear.2025.101176 (2025).

[54] Chai, Y. et al. Constrained CMIP6 projections indicate less warming and a slower increase in water availability across Asia. *Nat. Commun.* **13**, 4124 (2022). 35840591 10.1038/s41467-022-31782-7 PMC9287300

